# Use of Microwave Maceration in Red Winemaking: Effect on Fermentation and Chemical Composition of Red Wines

**DOI:** 10.3390/molecules27093018

**Published:** 2022-05-07

**Authors:** Raquel Muñoz García, Rodrigo Oliver-Simancas, María Arévalo Villena, Leticia Martínez-Lapuente, Belén Ayestarán, Lourdes Marchante-Cuevas, María Consuelo Díaz-Maroto, María Soledad Pérez-Coello

**Affiliations:** 1 Area of Food Technology, Faculty of Chemical Sciences and Technologies, Regional Institute for Applied Scientific Research (IRICA), University of Castilla-La Mancha, Avda, Camilo José Cela 10, 13071 Ciudad Real, Spain; raquel.munoz@uclm.es (R.M.G.); rodrigo.oliver@uclm.es (R.O.-S.); maria.arevalo@uclm.es (M.A.V.); soledad.perez@uclm.es (M.S.P.-C.); 2 Institute of Vine and Wine Sciences, University of La Rioja, La Rioja Government and CSIC, Finca La Grajera, Ctra, De Burgos Km 6, 26007 Logroño, Spain; leticia.martinez@unirioja.es (L.M.-L.); belen.ayestaran@unirioja.es (B.A.); 3 Regional Institute for Research and Agrifood and Forestry Development of Castilla La Mancha (IRIAF), IVICAM, 13700 Tomelloso, Spain; lmarchantec@jccm.es

**Keywords:** red wine, microwave maceration, grape yeasts, amino acids, polysaccharides

## Abstract

The objective of this study was to evaluate the effect of microwave treatment of crushed grapes on the yeast population of the must and on the development of alcoholic fermentation, as well as on the extraction of different compounds from the grapes such as polysaccharides and amino acids that can affect the organoleptic quality and stability of the wine. This study demonstrated for the first time the effect of the microwave treatment of grapes on native yeast species and their diversity, producing an increase in fermentation kinetics and a decrease in the lag phase. The microwave treatment produced a positive effect on the extraction of amino acids and polysaccharides from the grapes, resulting in significantly higher amounts of the main amino acids of the must and some major volatile compounds in the treated samples. The polysaccharides most affected by the microwave treatment were the PRAGs, the main polysaccharides liberated from grapes during the maceration.

## 1. Introduction

In the production of red wines, maceration is the most decisive stage of the process. During the maceration period, the extraction of compounds from the grape skin is favored, which will be essential to determine the quality of the wine. Several technologies have been applied with the aim of accelerating the extraction process; among them are thermovinification, cryomaceration, or flash release systems, but many of them involve a high energy cost [1,2]. In recent years the use of new technologies in winemaking such as ultrasound, microwave, or electric fields are emerging as environmentally friendly processes with high energy conversion efficiency [3]. The use of high-frequency ultrasound, recently authorized by the OIV, has proven to be an effective technique for increasing the extraction of phenolic compounds, varietal volatile compounds, and grape polysaccharides, considerably reducing maceration times [4,5,6,7].

In the food industry, microwave technology has been applied to reduce processing times and for food preservation [3]. Microwaves produce molecules with permanent dipoles to rotate in aliquid matrix, causing molecular movement by migration of ions. These effects produce internal superheating of cells, pressure increase, and dehydration. Additionally, conformational changes occur in membrane proteins that cause the opening of pores in the cell wall. All these effects facilitate the migration of compounds from grape to must [8].

Microwave treatment applied to the crushed grapes in the maceration phase has recently been used to reduce maceration times in red winemaking for different grape varieties, achieving rapid extraction of phenolic compounds, especially in unripe grapes [9,10,11,12,13,14]. Microwave maceration also increased the free and bound fraction of most varietal compounds in the must, obtaining wines with a greater fruity and floral aroma and allowing a reduction in SO_2_ levels [9,15]. However, the application of microwaves in wines during the ageing period increased the aromatic intensity of wood attributes and accelerated the ageing process [16,17,18].

Since the use of microwaves in the maceration phase is an effective technique in the extraction of phenolic or volatile compounds from the grapes, the same effect could be expected in improving the extraction of other compounds located in the grape skin, such as amino acids or polysaccharides, which are of great interest in the characteristics of the wine. In fact, the nitrogen compounds of grapes, especially assimilable amino acids and ammonium (YAN), play a priority role in the growth of yeasts, requiring minimum levels for the optimal development of alcoholic fermentation [19]. In addition, amino acids act as precursors of volatile compounds that can influence the aroma of wines, or as precursors of other compounds that can generate toxicity, such as biogenic amines [20,21].

The amino acid content of grapes can be affected by many factors such as climatic conditions, culture practices, or grape variety [20,21,22,23]. Likewise, winemaking techniques such as maceration time or type of yeast used in fermentation could have a great influence on the final content of amino acids in wines [24]. Carew et al., 2014 [25] observed an increase in the amount of total assimilable nitrogen in musts from microwave maceration, but no study has been carried out on the effect of this treatment on the amino acid fraction.

Polysaccharides are one of the main groups of macromolecules in red wines and play an important role in the stabilization of other molecules and in the perceived organoleptic properties of the beverage [26]. However, not all polysaccharides show the same behavior with respect to wines and their influence on wine will depend on their quantity and the type of polysaccharides [27].

Wine polysaccharides are primarily derived from grapes and yeasts. Polysaccharides from grapes include those rich in arabinose and galactose (PRAGs), comprising arabinans, arabinogalactans, and arabinogalactan-proteins (AGPs); rhamnogalacturonans type I and II (RG-I and RG-II), and homogalacturonans (HLs). Polysaccharides from yeast are constituted by mannoproteins (MPs), which are released by the yeast during the fermentation and aging process. Grape polysaccharides are extracted during maceration and pressing, requiring several days of maceration to achieve the desired extraction [28]. In fact, the time of maceration is one of the main factors affecting the polysaccharide content in wines [29]. Therefore, various treatments have been proposed to enhance the degradation of grape berry cell walls, increasing the polysaccharide extraction and reducing the maceration time [7,30,31]. Among these treatments, the application of microwaves to crushed grapes could be a promising technology to facilitate the extraction of polysaccharides because it has effective cell wall disruption abilities with short processing time [32].

However, when microwave treatment is applied in winemaking, its possible effect on the microbial population and fermentation must be considered. Microbial cells can be affected by microwaves, depending on the frequency and intensity of radiation; some authors have observed cell destruction while others have proposed a microbial growth [33,34]. Carew et al., 2014 [10] showed a significant reduction in native grape yeast populations and faster fermentation kinetics in wines from microwave treatment of crushed grapes. Endogenous yeasts play an important role in the first days of fermentation, since they can produce metabolites that will influence the final composition of the wine [35]. However, there are no specific studies on how microwave treatment affects the different species of autochthonous grape yeasts.

This study aimed to evaluate the effect of low-power microwave treatment with controlled temperature applied to crushed grapes before fermentation on the initial population of grape yeasts, the fermentation kinetics, and the chemical composition of the wine, especially in the content of amino acids and polysaccharides.

## 2. Results

### 2.1. Effect of Microwave Treatment of Crushed Grapes on the Must Biota

#### 2.1.1. Microorganism Isolation and Counts

To study the effect of microwave treatment on microbial population, samples of must from freshly crushed grapes were taken before starting fermentation to perform a count of total yeasts and bacteria. The counts obtained for yeasts and bacteria in non-treated must (4.93 ± 1.02 and 3.92 ± 0.89 log10 cfu/m, respectively) were like those obtained from microwave-treated must (5.03 ± 1.77 and 3.96 ± 1.10 log10 cfu/m), and no significant differences were found. The populations of bacteria and yeasts were as expected in fresh must when yeasts increase the viable cells while bacteria decrease them [36].

There are some studies about the inactivation of microorganisms by microwaves but very few when microwaves are used at low power during the grape maceration process. The mechanism by which microwaves contribute to additional microbial destruction, besides heat damage to cells, is still unclear. Although some studies support the efficiency of microwaves [37], others oppose the concept [38,39]. The destruction of various pathogens such as *E. coli* and *Staphylococcus aureus* has been observed under microwave treatment at high-power conditions [34]. However, microwaves have shown a resonant-like effect on the growth of some yeasts, including *Saccharomyces cerevisiae*, increasing the yeast growth, which was not dependent on the amount of absorbed energy from the microwaves [40].

In the oenological field, high-power microwave treatment has been applied to reduce microbial populations (lactic bacteria and Brettanomyces) in oak barrels by González-Arenzana et al. [41]. In addition, a decrease in yeast viability was found by Carew et al. 2014 [25] when using microwaves in grape maceration at 1500 W reaching 70 °C, although they observed an activation of the remaining yeasts (decrease in the lag phase of alcoholic fermentation on microwave-treated must).

The results of the present study showed no influence on the microorganism viability after microwave treatment of crushed grapes using 700 W and temperatures below 50 °C. More studies will be necessary to clarify the behavior of microorganisms after microwave treatment at different conditions of power, time, and temperature.

#### 2.1.2. Yeast Isolation, Identification, and Diversity Study

For genetic identification of yeast species by PCR-RFLP, twenty isolates from each must sample were selected. To confirm this identification a representative sample from each profile was used for genetic DNA sequencing. In most cases the PCR-RFLP patterns coincided with sequencing (90% of similarity). Some yeasts could not be found in the database and were also sequenced.

**Figure 1:**
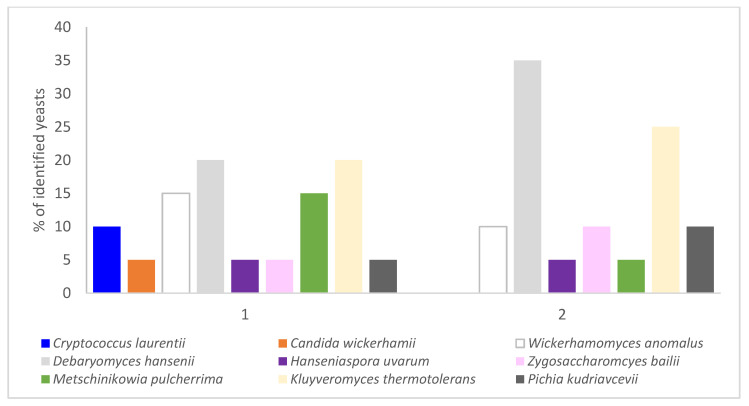
Percentage of yeast isolates from non-treated (control must) and microwave-treated musts (1 and 2, respectively).

Results showed that the 20 isolates from control musts (without microwave treatment) were catalogued in 9 distinct species: *Cryptococcus laurentii*, *Candida wickerhamii*, *Wickerhamomyces anomalus*, *Debaryomyces hansenii*, *Hanseniaspora uvarum*, *Zygosaccharomyces bailii*, *Metschinikowia pulcherrima*, *Kluyveromyces thermotolerans*, and *Pichia kudriavcevii*. Nevertheless, in the microwave-treated must, only 7 species were identified. The species *Cr. Laurentii* and *C. wickerhamii* were not found (Figure 1).

Most of the species identified agree with the native species usually reported by other studies in musts [36,42] with no presence of *Saccharomcyes* spp. As it has been reported above, the total yeast population was not affected by the microwave treatment, but there was a reduction in the number of species identified and their percentages were affected by the treatment. The species that mainly prevailed after microwave maceration were *D. hansenii* and *K. thermotolerans*, which increased their percentages from 20 to 35% and from 20 to 25% respectively.

In Table 1 the Simpson index and the percentage of genetic diversity are shown. The first indicates the contribution to the diversity of each species. The higher the number of isolates of each species, the lower is its contribution to the diversity. The yeasts that favored the diversity in non-treated must (control must) were *C. wickerhamii*, *H. uvarum*, *Z. bailii*, and *P. kudriavcevii*, while in microwave-treated must were *H. uvarum* and *M. pulcherrima*. However, the major percentage and the lowest contribution to diversity were obtained in the case of *D. hansenii* and *K. thermotolerans* for both samples.

This study demonstrates for the first time the effect of microwave treatment in crushed grapes on native yeast species and their diversity. The role of some non-Saccharomyces genera such as *Hanseniaspora, Pichia*, *Metschnikowia*, or *Schizosaccharomyces* among others in winemaking is very important, even when *Saccharomcyes cerevisiae* starter cultures are employed [43]. They are naturality present in the first stage of the process and can improve the sensory profile of wines because they affect aroma, color, and mouthfeel by production of aromatic esters and liberation compounds of interest after lysis [35,44]. In fact, one of the possible criteria for the selection of a starter culture is the non-production of killer toxins for ensuring the prevalence of the autochthonous biota at the beginning of the fermentation.

However, many of the different yeast capabilities involved in vinification, regarding both improvement of the quality of the products and the sensitivity of the treatments, are strain-dependent, as occurs with other microbial characteristics [45,46].

### 2.2. Effect of Microwave Treatment of Crushed Grapes on Fermentation Kinetics and Basic Chemical Composition of Musts and Wines

**Figure 2:**
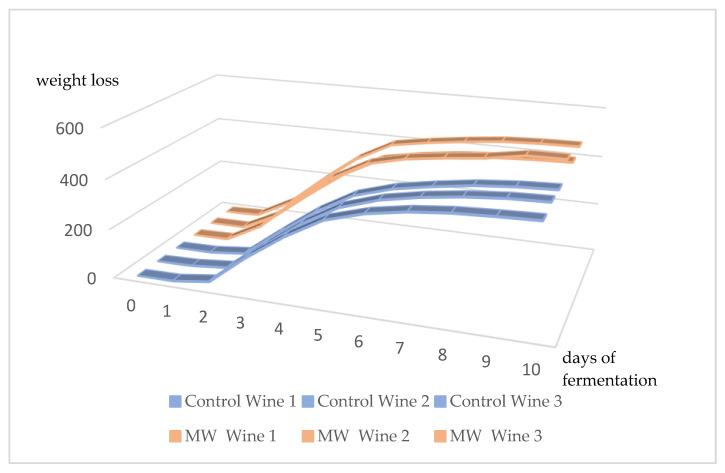
Alcoholic fermentation kinetics measured by the weight loss in control wines and wines from microwave treatment (triplicates of fermentation are shown).

Figure 2 shows the evolution of the alcoholic fermentation, measured by the weight loss of the triplicates. Fermentation from microwave-treated grapes presented a shorter period of lag phase and a higher yield. This fact has been observed by other authors in wines from different grape varieties using microwaves in the maceration phase [10,25]. Microwave treatment achieves greater extraction of grape compounds used as nutrients by yeast, which could accelerate the fermentation process. Additionally, morphological and metabolic changes in yeast cells induced by MWs could cause activation of the yeasts after treatment [34].

Therefore, the microwave treatment in maceration not only allows correct development of the alcoholic and malolactic fermentation [9], but according to our experience the effect caused by MWs could also be desirable for wine fermentation.

The results of the basic composition of musts and wines with and without microwave treatment are shown in Table 2. In general, the basic chemical composition of the wines was slightly influenced by the microwave treatment as has been evidenced in wines from other grape varieties [11,12]. Only some compounds from alcoholic fermentation, such as succinic acid and glycerol, presented significantly higher amounts in wines from MW treatment, which may be related to more efficient fermentation kinetics (Figure 1). Both organic acids and glycerol play an important role in the organoleptic characteristics of wine.

However, the total polyphenol index (TPI) suffered a very important increase due to the microwave treatment, especially in the must. Other authors have also described a significant increase in total phenolics extraction and other parameters related to color (anthocyanins, flavonols, tannins, etc.) in wines when applying high-power microwaves in grape maceration [10,11,25]. In our case, the power and times used were lower to avoid an increase in temperature. Despite this, the microwave treatment caused a significant increase in the content of total polyphenols in the wine, which undoubtedly has important implications for its color and evolution.

### 2.3. Effect of Microwave Treatment of Crushed Grapes on Amino Acid Composition of Must and Wines

Table 3 shows the content of assimilable amino acids and ammonium of musts and wines (control and microwave-treated samples). The amino acids of the must, together with the ammonium, are the main source of nitrogen used by the yeasts for their growth. Higher amino acid content can influence yeast growth and then increases the fermentation rate and yeast metabolite production [19].

However, amino acids are considered precursors of volatile compounds formed by yeasts during alcoholic fermentation. The relationship between the availability of nitrogenous sources in the grapes and the production of volatile compounds in the wine has been extensively studied. Wines poor in nitrogen give rise to wines with less intense aromas due to the lower production of volatile compounds [47].

The amounts of ammonium in the must are small and it is initially consumed by yeasts; together with the amino acids it contributes to the yeast assimilable nitrogen (YAN). The total assimilable nitrogen of the must was significantly affected by the microwave treatment. This effect was also observed by other authors in the Pinot Noir variety subjected to high-power microwaves during maceration [25].

Most of the must amino acids were increased due to microwave treatment. Among them are the major amino acids: alanine and GABA, which were widely assimilated by yeasts. The total amino acid content of musts presented a reduction between 72% in the control wines and 75% in wines from MW treatment, which implies a greater consumption by the yeasts during the alcoholic fermentation in these samples.

Most amino acids remained higher in wines from MW treatment, although the profile changed, with the main amino acids being tyrosine, glutamic acid plus glutamine and asparagine. Significant differences due to MW treatment were observed in all of them.

A low consumption of sulfur amino acids (methionine, cysteine, and ornithine) was detected, which may affect the production of compounds derived from them such as methionol or hydrogen sulfide [48]. Likewise, a small increase was observed in the content of some amino acids in the wine with respect to the must, such as lysine and glycine. This may be due to their release into the medium at the end of fermentation by yeast secretion or during the autolysis of dead yeasts.

### 2.4. Effect of Microwave Treatment of Crushed Grapes on Major Volatile Compounds in Wines

Table 4 shows the concentrations of major volatile compounds formed during alcoholic fermentation in the control wines and in wines from microwave treatment. Acetaldehyde is the major aldehyde from the carbohydrate metabolism of yeasts. High concentrations of this compound can generate an unpleasant aroma in wines, being found in low amounts in both control and microwave-treated wines.

Higher alcohols include propanol, isobutanol, isoamyl alcohols (2-methyl-1-butanol and 3-methyl-1-butanol), and 2-phenylethanol. These compounds can be formed during alcoholic fermentation from some musts’ amino acids (valine, leucine, isoleucine, threonine, and phenylalanine) following the Ehrlich pathway, or from sugar metabolism, which is usually the major pathway [19].

Wines from microwave maceration showed higher concentrations of propanol, isoamyl alcohols, and 2-phenylethanol than control wines. In general, high amounts of higher alcohols are not desirable in wines since they provide unpleasant odors (medicinal, solvent-like), although they have high odor thresholds so the influence on the overall aroma of the wine is usually limited. On the contrary, 2-phenylethanol, with a pleasant aroma of roses, was found above its odor threshold in the microwave-treated samples [49].

Regarding esters, only isoamyl acetate, characterized by its pleasant banana aroma, showed a small increase in wines from microwave treatment, which could positively influence the final aroma of these wines.

Fortunately, no effect of microwave maceration was observed on the extraction of methanol, a compound located in grape skins with harmful effects on health.

The positive effect of microwave treatment in maceration on the content of minor volatile compounds from fermentation has previously been observed in Cabernet Sauvignon wines, especially in the content of some esters, acetates, and alcohols [15]. This effect may be related to a higher extraction of nutrients during maceration and more efficient fermentation kinetics, as seen above. However, in Tempranillo wines a decrease in the amounts of higher alcohols and a significant increase in some fatty acids was observed using MW in grape maceration [18], so there could be different behavior depending on the grape variety and the MW treatment conditions.

### 2.5. Effect of Microwave Treatment of Crushed Grapes on Polysaccharide Composition of Must and Wines

Table 5 shows the monosaccharide composition of the main polysaccharides present in musts and wines. Glucose was the predominant glycosyl residue detected in must samples, although its content was higher in microwave-treated must and wine and no significant differences among samples were found. According to the literature, glucose is the prevalent sugar in both the skin and pulp cell walls of grape berries [28,50], because it is the main component of major structural polysaccharides from the grape cell walls.

After glucose, the most prevalent glycosyl residues were galactose, arabinose, and galacturonic acid, which are components of must pectic PRAGs, such as galacturonans, galactans, arabinogalactans, arabinogalactan proteins and arabinans, and homogalacturonans [51]. Rhamnose and glucuronic acid were also detected in smaller amounts. The identification of rare sugars such as apiose, 2-*O*-methyl-fucose, and 2-*O*-methyl-xylose, indicated the presence of rhamnogalacturonan type II (RG-II) [52]. The presence of xylose indicated that traces of hemicelluloses might be solubilized from grape berry cell walls [53].

Except for galactose, a significantly higher content of individual glycosyl residues in the microwave-treated must with respect to the control was observed. 2-*O*-methyl-xylose and Kdo were the glycosyl residues that showed the most different content between MW-treated and non-treated must. Consequently, the total content of pectic monosaccharides coming from the grape skin was higher in the must treated with microwaves, while these differences were not significant in the case of wine.

Mannose in must could come from mannoproteins (MPs) of endogenous yeast cell walls [28] or from mannans or xyloglucans [54,55,56] so its amount in wines increases substantially.

The Ara/Gal ratio is characteristic of the wine PRAG composition [56,57]. The Ara/Gal ratios in musts were similar to those described in the literature for white and red musts [7,51]. MW-treated must showed a significant higher Ara/Gal ratio than control must, suggesting a larger release of arabinose or polysaccharides rich in arabinose arising from the ramified or hairy region of the pectic framework in the case of treated musts.

**Figure 3:**
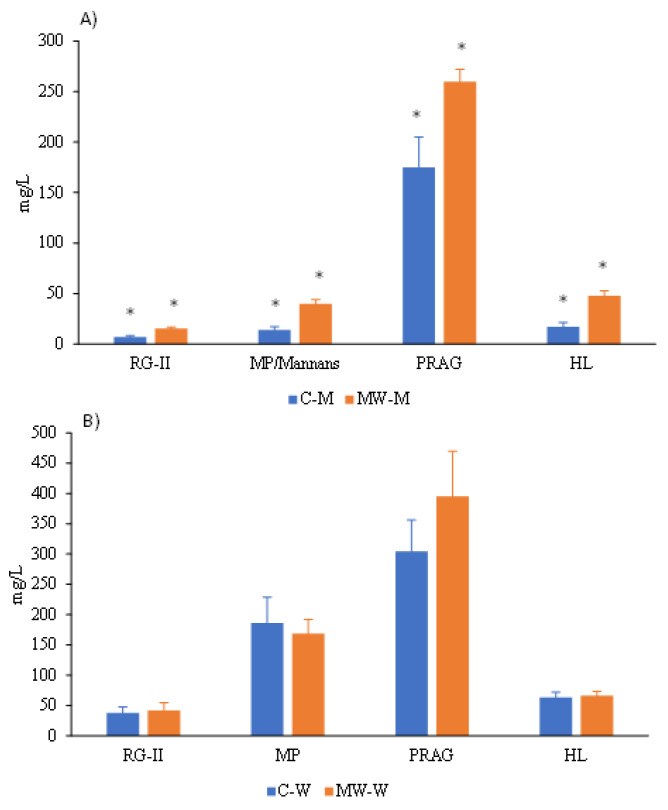
Concentration of rhamnogalacturonan type II (RG-II), mannoproteins (MPs) or mannans, polysaccharides rich in arabinose and galactose (PRAGs), and homogalacturonans (HLs) in musts (**A**). Concentration of rhamnogalacturonan type II (RG-II), mannoproteins (MPs), polysaccharides rich in arabinose and galactose (PRAGs), and homogalacturonans (HLs) in wine (**B**). C-W: control must. MW-W: must from microwave-treated grapes. C-W: control wine. MW-W: wine from microwave-treated grapes. Values with (*) show significant differences according to the *t*-test (*p* < 0.05).

Regardless of the treatment, PRAGs were the main polysaccharides liberated from crushed and pressed grapes, and they accounted for more than 70% of total must polysaccharides (Figure 3).

Microwave treatment improved the breakdown of cell walls of crushed grapes, thereby it significantly increased the content of PRAGs, RG-II, HLs and mannans/MPs in musts.

The most marked impact of the winemaking process was an increase in pectic monosaccharides and a significant decrease in glucose content. Microwave effectiveness in wines was less relevant than in the must. Although no significant differences were shown between the control and treated wines in the content of PRAGs, RG-II, HLs and MPs (Figure 3), in the case of PRAGs an increase was observed in wines from microwave treatment.

## 3. Materials and Methods

### 3.1. Grape Samples

*Cabernet Sauvignon* red grapes were kindly provided by the Institute of Vine and Wine of Castilla-La Mancha (IVICAM, Tomelloso, Ciudad Real, Spain).

### 3.2. Microorganism Isolation and Counts

Sampling of treatment and non-treatment musts was performed. For isolation of yeasts, samples and/or serial dilutions were streaked onto YPD agar (1% yeast extract, 2% peptone, and 2% glucose) with added tetracycline (25 μL/mL) and sodium propionate (0.25 g/L) to inhibit bacteria and mold growth, respectively. Plates were incubated at 28 °C for 48 h and counts were carried out.

The same process was carried out for bacteria counting using PCA agar (5% yeast extract, 5% Triptone, 2.5% glucose) with added cycloheximide (0.1 g/L) and sodium propionate (0.25 g/L) for inhibition of yeasts and molds, respectively, and incubating for 48 h at the same temperature. Counts were performed in duplicate.

#### 3.2.1. Yeast Isolation and Purification

Twenty isolates were selected from each sample trying to pick the different morphologies. Yeasts were streaked on YPD agar and were incubated at 28 °C for 48 h. All the isolates were preserved in 15% glycerol at −80 °C until analysis.

#### 3.2.2. Yeast Isolation and Identification

PCR-RFLP: Genetic species identification of yeasts was carried out using the PCR-RFLP technique by amplifying the ITS1 and ITS2 sequences surrounding the 5.8S rRNA gen with the ITS1 (50TCCGTAGGTGAACCTGCGG30) and ITS4 (50TCCTCCGCTTATTGATATGCC30) primers [58]. Amplification was carried out on a Perkin-Elmer 2400 thermal cycler. PCR conditions were an initial denaturation at 95 °C/50 and 35 cycles with the subsequent conditions: 95 °C/10 (denaturation), 555 °C/10 (hybridization), 72 °C/103,000 (extension) and a final cycle at 72 °C/100 (final extension). PCR products were digested with the restriction endonucleases Hinf I, Hae III, and Cfo I [59]. Hpa II and Dde I enzymes were used to identify *Saccharomyces* spp. species. PCR products and their restriction fragments were separated on 2% agarose gel and were visualized by the gel Green (6×) in a gel documentation system. Sizes were estimated by comparison against a 100 bp DNA length standard. The yeast ID database (CECT, University of Valencia and CSIC, Valencia, Spain) was used to identify the profiles.

Genetic DNA sequencing: With the aim of confirming the species found, a representative from each profile identified from PCR-RFLP analysis was selected together with non-identified isolates, and the D1/D2 domain was sequenced from the 26S rRNA, amplified by NL1 and NL4 primers. For results with <99% identity, the ITS region was also sequenced using ITS1 and ITS4 primers (Laboratory of Instrumental Techniques, University of León). Once the sequences were obtained, the identity was searched in the BLAST database (GenBank, USA National Library of Medicine).

#### 3.2.3. Diversity Study of the Yeasts

Simpson’s Diversity Index was calculated in order to measure the contribution to the diversity of each species, considering the number of species present and the relative abundance of each among the total microorganisms found. The following equation was applied: *D* = 1 − (Σ*n*(*n* − 1)/*N*(*N* − 1)), where *n* is the total number of organisms of a species and *N* is the total number of organisms of all species in the same sample. The value of *D* ranges between 0 (no diversity) and 1 (infinite diversity).

The genetic diversity percentage was calculated to evaluate the genetic diversity in the species identified by comparing the number of strains detected with the number of yeast isolates per species. It was calculated as follows: % = (*ns/ni*)100, where *ns* is the total number of strains per species and *ni* is the total number of strains in the sample. The calculated percentage ranges were between >0 and 100% and the highest values indicate the highest strain variety.

### 3.3. Winemaking Process

*Cabernet Sauvignon* red grapes were harvested at 24.9 °Brix. After destemming and crushing, samples were divided into two batches (60% must and 40% skins for each one) and sulfited with 50 mg/L of potassium metabisulfite. One batch was immediately moved to a chamber at 22 °C (±2 °C) without any treatment and used as control. The other batches (MW) were microwave-macerated for 12 min at 700 W using a domestic LG microwave oven (LG electronics). The temperature of the batch prior to the treatment was 14 °C. To avoid an increment up to 50 °C, the microwave treatment was realized at 4 min intervals. At the end of each time interval samples were stirred, and their temperatures were evaluated using a solid stem thermometer. All treatments were executed in triplicate. Then, microwave-treatment batches were moved to a 22 °C (±2 °C) chamber for sampling, yeast inoculation, and fermentation.

Alcoholic fermentation was realized in 10 L glass containers that were not filled more than 60%, using *Saccharomyces cerevisiae* (CECT no. 10835) as the starter culture, at 22 °C (±2 °C). Two punching-downs were carried out every day and after three days of maceration, the skins were separated from the must to continue fermentation. The evolution of the fermentation was controlled by weight loss, and it was considered finished when the container weight remained stable. Then, the density and glucose and fructose levels of wines were determined. Finally, wines were decanted, cold stabilized at −5 °C, filtered and bottled. Wines were adjusted to 25 mg/L of free SO_2_ before bottling.

### 3.4. Conventional Analysis

Conventional analysis (density, alcoholic degree, °Brix, pH, total and volatile acidity, free SO_2_, glucose and fructose, glycerin, and organic acids (malic, lactic, citric, tartaric, and succinic acids and TPI) were determined by official analytical methods established in the International Organization of Vine and Wine (OIV, 2020).

### 3.5. Amino Acid Analysis

The determination of amino acids was carried out using the method described by Gómez-Alonso et al. [60] with some modifications. Previously, the samples were derivatized by mixing 1 mL of wine with 1.75 mL of 1 M borate buffer (pH = 9), 30 μL of diethylethoxymethylenemalonate (DEEMM), and 750 μL of methanol in a screw-cap test tube for 30 min in an ultrasound bath. To allow complete degradation of excess DEEMM and reagent by-products, the mixture was heated at 70 °C for 2 h.

HPLC equipment was used to perform the analyses with a diode array detector (Agilent, Model 1100; Agilent Technologies, Inc. Santa Clara, CA, USA). The chromatographic separation was carried out on an ACE HPLC column (5 C18-HL), with a particle size of 5 μm (250 mm × 2.1 mm), with the gradient shown in Table 1 (phase A: 25 mM acetate buffer, pH = 5.8 with 0.02% sodium azide; phase B: methanol; phase C: acetonitrile), and a flow rate of 0.9 mL min^−1^. For detection, a photodiode array detector was used, monitored at 280 and 269 nm. Compounds were identified and quantified using the corresponding standards (Sigma-Aldrich Chemie GmbH, Steinheim, Germany).

### 3.6. Major Volatile Compound Analysis

The GC/MS Focus-ISQ chromatograph (Thermo Scientific, Milan, Italy) was used to analyze the major volatile compounds. To prepare the sample, 100 μL of wine was blended with 100 μL of 2-pentanol-4-methyl used as internal standard (41.55 mg/L) and 1 mL of Mili-Q water. Later, 1 μL of sample was injected in split mode (1/25) onto a BP-21 (SGE) column (60 m × 0.32 mm × 0.25 μm) of FFAP (nitroterephthalic acid-modified polyethylene glycol (TPA) phase. The carrier gas used was helium (constant flow rate of 1.2 mL/min). The injector temperature was 195 °C and the oven temperature was programmed starting at 32 °C for 2 min, ramped to 5 °C/min to 120 °C, and rising up 75 °C/min to 190 °C (maintained for 18 min). The MS was operated in electron impact mode: electron energy 70 eV, ion source temperature 250 °C. Compound identification was achieved by comparing with commercial standards (Sigma-Aldrich Chemie GmbH, Steinheim, Germany) and calibration curves of each standard were made by quantification purpose.

### 3.7. Identification and Quantification of Monosaccharides by GC–MS

Must and wine polysaccharides were recovered by precipitation after ethanolic dehydration as previously described [27,61]. The monosaccharide composition was determined by GC–MS of their trimethylsilyl-ester O-methyl glycosyl residues obtained after acidic methanolysis and derivatization as previously described [27]. GC was controlled by ChemStation software and equipped with a 7653B automatic injector consisting of an Agilent 7890A gas chromatograph (Agilent Technologies, Waldbronn, Germany) coupled to a 5975C VL quadrupole mass detector (MS). The content of each polysaccharide family was estimated from the concentration of individual glycosyl residues, which are characteristic of structurally identified must and wine polysaccharides [52,61].

PRAGs were estimated from the sum of galactosyl, arabinosyl, rhamnosyl, and glucuronosyl residues; all the mannose content in wines was attributed to yeast mannoproteins; the RG-II content was calculated from the sum of its diagnostic monosaccharides, which represent approximately 25% of the RG-II molecule. Taking into account the molar ratios of the RG-II (1 residue of 2-O-methyl fucose, 3.5 rhamnose, 2 arabinose, 2 galactose, 1 glucuronic acid, and 9 galacturonic acid), the remaining part was attributed to the presence of PRAGs in the case of rhamnose, arabinose, galactose, and glucuronic acid. The remaining galacturonosyl residues were used to estimate the content of homogalacturonans (HLs) [27,62].

### 3.8. Statistical Analysis

Statistical analysis was executed using the IBM SPSS statistics v.24.0 for Windows statistical package. Student’s t-test was used to find significant differences between samples.

## 4. Conclusions

The application of microwaves in crushed grape maceration did not modify the population of yeasts and total bacteria in the must, although it had an important effect on the biodiversity of the grape biota, producing a reduction in the number of species identified. Additionally, a reduction in the lag phase and more efficient fermentation kinetics were observed in wines from microwave treatment. The basic composition of the treated wine was little affected, but samples from microwave treatment showed a higher total polyphenol index, and higher content of major amino acids and total assimilable nitrogen. Some volatile compounds that could influence the aroma of wines, such as 2-phenylethanol and isoamyl acetate, were also increased by MW treatment.

The effect of microwave grape treatment on polysaccharide composition was more evident in musts than in wines, mainly in the case of pectic monosaccharides. Likewise, the PRAGs, the main polysaccharides liberated from crushed grapes, showed higher amounts in musts when microwaves were applied before maceration.

According to the results obtained, the microwave treatment of crushed grapes is proposed as a very interesting technique to increase the extraction of compounds from the grape, such as amino acids and polysaccharides, which may play an important role in the organoleptic quality and stability of the wine. In addition, the effect of this treatment on the microbial population of the must and the development of fermentation must be considered. In any case, it would be necessary to carry out additional experiments with different grape varieties and degrees of maturation to corroborate these results.

## Figures and Tables

**Table 1:** Diversity study of the must yeasts: D (Simpson index) and % diversity of each species.

Species	Control Must		MW-Treated Must	
	D	% Diversity	D	% Diversity
*Cryptococcus laurentii*	0.95	10	-	0
*Candida wickerhamii*	1	5	-	0
*Wickerhamomyces anomalus*	0.89	15	0.95	10
*Debaryomyces hansenii*	0.84	20	0.68	35
*Hanseniaspora uvarum*	1	5	1	5
*Zygosaccharomcyes bailii*	1	5	0.95	10
*Metschinikowia pulcherrima*	0.89	15	1	5
*Kluyveromyces thermotolerans*	0.84	20	0.84	25
*Pichia kudriavcevii*	1	5	0.95	10

**Table 2:** Basic chemical composition parameters of musts and wines (control samples and samples from microwave treatment).

Parameter	Control Must	MW Must	Control Wine	MW Wine
	MEAN ± SD	Mean ± SD	Mean ± SD	Mean ± SD
Ethanol (% *v*/*v*)	-	-	13.8 ± 0.2	14.1 ± 0.2
pH	3.45 ± 0.02	3.55 ± 0.03	3.09 ± 0.06	3.16 ± 0.04
Titratable acidity (g/L)	3.81 ± 0.03	3.66 ± 0.03	8.48 ± 0.61	7.88 ± 0.10
Volatile acidity (g/L)	-	-	0.06 ± 0.02	0.09 ± 0.01
Glucose + fructose (g/L)	251.06 ± 17.59	244.27 ± 2.37	5.82 ± 0.99	5.02 ± 1.57
Malic acid (g/L)	0.98 ± 0.07	0.99 ± 0.05	1.59 ± 0.06	1.61 ± 0.03
Citric acid (g/L)	>0.1	>0.1	0.28 ± 0.00	0.30 ± 0.00
Tartaric acid (g/L)	2.70 ± 0.26	2.60 ± 0.06	2.18 ± 0.04	1.79 ± 0.03
Succinic acid (g/L)	-	-	1.69 ± 0.05 *	1.87 ± 0.05 *
Lactic acid (g/L)	0.07 ± 0.01	0.06 ± 0.01	0.10 ± 0.02	0.13 ± 0.02
Glycerol (g/L)	-	-	9.67 ± 0.35 *	10.43 ± 0.20 *
TPI (mg/L of galic acid)	439.94 ± 6.32 *	915.09 ± 11.24 *	2211.82 ± 5.23 *	2472.42 ± 6.30 *

Values with (*) show significant differences according to the *t*-test (*p* < 0.05).

**Table 3:** Amino acid composition and ammonium content (mg/L) of musts and wines (control samples and samples from microwave treatment).

Compound	Control Must	MW Must	Control Wine	MW Wine
	Mean ± SD	Mean ± SD	Mean ± SD	Mean ± SD
Aspartic acid	6.36 ± 0.31 *	8.53 ± 0.14 *	1.45 ± 0.53	1.34 ± 0.36
Glutamic Acid + Glutamine	23.70 ± 0.92 *	27.50 ± 0.80 *	12.91 ± 0.37 *	15.39 ± 0.38 *
Asparagine	27.60 ± 2.55 *	36.34 ± 3.72 *	13.78 ± 0.13 *	15.89 ± 0.30 *
Serine + OH-Proline	19.51 ± 3.56	16.49 ± 0.07	1.62 ± 0.11 *	2.00 ± 0.08 *
Histidine	13.93 ± 1.86	15.26 ± 2.10	3.33 ± 0.11 *	3.66 ± 0.07 *
Glycine	1.14 ± 0.07	1.22 ± 0.08	2.40 ± 0.11 *	2.77 ± 0.17 *
Threonine	18.55 ± 0.80 *	21.46 ± 0.13 *	0.37 ± 0.03	0.30 ± 0.06
*β*-Alanine + Arginine	8.41 ± 1.84	8.82 ± 0.33	2.77 ± 0.07	2.76 ± 0.04
L-Alanine	49.80 ± 2.92 *	71.62 ± 4.75 *	2.89 ± 0.12 *	3.17 ± 0.03 *
GABA	32.23 ± 2.96 *	45.38 ± 0.45 *	2.11 ± 0.03	2.24 ± 0.12
Tyrosine	34.69 ± 2.74	37.46 ± 0.56	24.55 ± 0.31 *	27.64 ± 0.06 *
Valine	9.19 ± 0.68 *	12.45 ± 0.70 *	0.56 ± 0.19	0.61 ± 0.21
Methionine	0.63 ± 0.38	0.55 ± 0.15	1.10 ± 0.64	1.55 ± 0.56
Cysteine	0.46 ± 0.12	1.26 ± 0.65	0.50 ± 0.15	0.58 ± 0.24
Tryptophan	1.82 ± 0.74	1.90 ± 1.06	0.45 ± 0.22	0.69 ± 0.31
Isoleucine	4.41 ± 0.33 *	5.38 ± 0.29 *	0.41 ± 0.08	0.43 ± 0.12
Phenylalanine	3.58 ± 0.48 *	4.97 ± 0.58 *	0.52 ± 0.12	0.43 ± 0.03
Leucine	6.16 ± 1.17	6.16 ± 0.05	0.60 ± 0.08	0.71 ± 0.15
Ornithine	1.35 ± 0.04 *	2.22 ± 0.14 *	0.66 ± 0.04 *	0.80 ± 0.04 *
Lysine	1.00 ± 0.17 *	0.36 ± 0.47 *	2.06 ± 1.26	2.19 ± 0.50
Ammonium	5.81 ± 6.23	7.46 ± 9.36	0.46 ± 0.08 *	0.25 ± 0.04 *
**∑ Amino acids and ammonium**	270.33 ± 3.28 *	332.81 ± 5.24 *	75.49 ± 2.56 *	85.42 ± 0.11 *

Values with (*) show significant differences in the same winemaking stage according to the *t*-test (*p* < 0.05).

**Table 4:** Major volatile composition (mg/L) of control wines and wines from microwave treatment.

Volatile Compounds	Control Wine	MW Wine
	Mean ± SD	Mean ± SD
Acetaldehyde	57.06 ± 12.13	47.45 ± 6.64
Methanol	38.84 ± 4.32	35.97 ± 2.94
Propanol	102.18 ± 18.92 *	165.72 ± 30.11 *
Isobutanol	60.69 ± 14.34	49.42 ± 14.60
Isoamyl alcohols	559.07 ± 5.52 *	573.70 ± 30.36 *
2-Phenylethanol	17.76 ± 0.25 *	30.90 ± 0.70 *
Ethyl acetate	44.39 ± 11.45	51.31 ± 4.46
Ethyl butyrate	0.24 ± 0.03	0.28 ± 0.07
Isoamyl acetate	3.53 ± 0.57 *	4.39 ± 1.28 *
Ethyl lactate	3.80 ± 0.23	3.30 ± 0.45

Values with (*) show significant differences according to the *t*-test (*p* < 0.05).

**Table 5:** Monosaccharide composition (mg/L) of polysaccharides in musts and wines (control samples and samples from microwave treatment).

Parameter ^a^	Control Must	MW Must	Control Wine	MW Wine
	Mean ± SD	Mean ± SD	Mean ± SD	Mean ± SD
2-*O*-CH_3_-Fucose	0.91 ±0.24 *	1.44 ±0.14 *	4.24 ± 0.95	4.74 ± 0.92
2-*O*-CH_3_-Xylose	0.31 ± 0.01 *	1.18 ± 0.15 *	1.94 ± 0.46	2.31 ± 0.35
Apiose	0.35 ± 0.03 *	0.42 ± 0.02 *	0.93 ± 0.05	2.19 ± 1.44
Kdo	0.15 ± 0.08 *	0.80 ± 0.12 *	2.33 ± 1.05	1.19 ± 0.47
Galactose	123.67 ± 16.61	143.83 ± 1.65	225.58 ± 36.21	285.25 ± 35.17
Arabinose	39.07 ± 10.18 *	85.90 ± 8.70 *	75.12 ± 10.88	79.01 ± 12.70
Rhamnose	13.30 ± 4.59 *	24.66 ± 0.52 *	22.08 ± 3.31	32.94 ± 18.84
Galacturonic acid	25.32 ± 6.33 *	60.83 ± 4.95 *	101.20 ± 17.68	109.09 ± 15.09
Glucuronic acid	6.13 ± 0.67 *	16.82 ± 1.75 *	15.65 ± 8.80	35.60 ± 15.80
Fucose	1.15 ± 0.64 *	4.05 ± 1.45 *	2.14 ± 1.08	2.60 ± 1.30
**^1^ ΣPectic monosaccharides**	210.34 ± 39.38 *	339.93 ± 19.32 *	**451.21 ± 80.47**	**554.92 ± 102.08**
Xylose	6.35 ± 1.82 *	15.49 ± 2.70 *	7.42 ± 1.76	12.10 ± 4.14
Glucose	1280.92 ± 384.28	1765.80 ± 618.03	49.02 ± 11.54	60.94 ± 16.70
Mannose	13.92 ± 3.20 *	39.70 ± 4.14 *	186.26 ± 42.64	168.31 ± 23.50
**Ara/Gal**	0.37 ± 0.05 *	0.72 ± 0.06 *	0.40 ± 0.01 *	0.33 ± 0.01 *

^a^ Kdo: 2-keto-3-deoxyoctonate ammonium salt. ^1^ ΣPectic monosaccharides: as sum of 2-*O*-CH_3_-fucose, 2-*O*-CH_3_-xylose, apiose, Kdo, galactose, arabinose, rhamnose, galacturonic acid, glucuronic acid, and fucose. Values with (*) show significant differences according to the *t*-test (*p* < 0.05).

## Data Availability

Not applicable.
